# A Metabolic Probe-Enabled Strategy Reveals Uptake and Protein Targets of Polyunsaturated Aldehydes in the Diatom *Phaeodactylum tricornutum*

**DOI:** 10.1371/journal.pone.0140927

**Published:** 2015-10-23

**Authors:** Stefanie Wolfram, Natalie Wielsch, Yvonne Hupfer, Bettina Mönch, Hui-Wen Lu-Walther, Rainer Heintzmann, Oliver Werz, Aleš Svatoš, Georg Pohnert

**Affiliations:** 1 Bioorganic Analytics, Institute for Inorganic and Analytical Chemistry, Friedrich Schiller University, Jena, Germany; 2 Department Mass Spectrometry/Proteomics, Max Planck Institute for Chemical Ecology, Jena, Germany; 3 Department of Pharmaceutical and Medicinal Chemistry, Institute of Pharmacy, Friedrich Schiller University, Jena, Germany; 4 Biomedical Imaging, Department Microscopy, Leibniz Institute of Photonic Technology e.V., Jena, Germany; 5 Institute for Physical Chemistry, Abbe Center of Photonics, Friedrich Schiller University, Jena, Germany; Universitat Bremen, GERMANY

## Abstract

Diatoms are unicellular algae of crucial importance as they belong to the main primary producers in aquatic ecosystems. Several diatom species produce polyunsaturated aldehydes (PUAs) that have been made responsible for chemically mediated interactions in the plankton. PUA-effects include chemical defense by reducing the reproductive success of grazing copepods, allelochemical activity by interfering with the growth of competing phytoplankton and cell to cell signaling. We applied a PUA-derived molecular probe, based on the biologically highly active 2,4-decadienal, with the aim to reveal protein targets of PUAs and affected metabolic pathways. By using fluorescence microscopy, we observed a substantial uptake of the PUA probe into cells of the diatom *Phaeodactylum tricornutum* in comparison to the uptake of a structurally closely related control probe based on a saturated aldehyde. The specific uptake motivated a chemoproteomic approach to generate a qualitative inventory of proteins covalently targeted by the α,β,γ,δ-unsaturated aldehyde structure element. Activity-based protein profiling revealed selective covalent modification of target proteins by the PUA probe. Analysis of the labeled proteins gave insights into putative affected molecular functions and biological processes such as photosynthesis including ATP generation and catalytic activity in the Calvin cycle or the pentose phosphate pathway. The mechanism of action of PUAs involves covalent reactions with proteins that may result in protein dysfunction and interference of involved pathways.

## Introduction

Oceans accommodate numerous coexisting microalga species in the plankton. Their community is shaped by different factors including nutrient limitation, predation and chemical signaling. Diatoms, a class of unicellular algae, are key players in the marine food web as they are responsible for about 40% of global marine primary productivity [1]. Some diatom species release biologically active metabolites as mediators of interactions. An intensively studied compound class in this context are oxylipins, which derive from the oxidative transformation of polyunsaturated fatty acids [2]. Of considerable interest among oxylipins are polyunsaturated aldehydes (PUAs), which have been reported to mediate various inter- and intraspecific interactions (reviewed in [2–5]). 2,4-Decadienal (DD) is the best studied metabolite of the group of PUAs, with attributed roles in grazer defense [6], allelophathy [7], cell to cell signaling [8], antibacterial activity [7,9] and bloom termination initiation [10,11]. PUA-mediated allelopathy [5,7,12,13] is impairing different phyla regarding growth and physiological performance. Sensitivity against PUAs has been reported for the prymnesiophyte *Isochrysis galbana* [7], the chlorophyte *Dunaliella tertiolecta* [7] as well as the centric diatom *Thalassiosira weissflogii* [14]. A synchronized release of PUAs from intact *Skeletonema marinoi* cells transiently before the culture changes to the decline phase supports the idea that PUAs play a role as infochemicals in mediating bloom termination [10]. Despite the well-documented biological functions of PUAs, their mechanism of action and their molecular targets are almost unknown [3,4]. Only few impaired biological processes and functions are recognized mainly involving disruption of intracellular calcium signaling, cytoskeletal instability and induction of apoptosis (reviewed in [2–4]).

PUA activity is structure-specific, since saturated aldehydes, like decanal that lack the conjugated α,β,γ,δ-unsaturated aldehyde motive of PUA, are not active [15,16]. Conjugated unsaturated aldehydes are reactive compounds belonging to the class of Michael acceptors. They act as electrophiles and react with proteins [17,18] and DNA [19–21]. Model investigations revealed that DD covalently modifies proteins by formation of imines (Schiff bases), pyridinium adducts and 1,4-addition products with nucleophiles [17,18]. Thus, proteins are putative targets of the electrophilic PUAs. PUAs also react with DNA resulting in apoptosis in copepods (reviewed in [22]). In algae [7], sea urchin embryos [23] and copepod embryos and nauplii [6,24] DNA laddering and chromatin dispersal or complete DNA fragmentation and dislocation is observed after PUA exposure.

The diatoms *Phaeodactylum tricornutum* [25] and *Thalassiosira pseudonana* [26] have emerged as model organisms since these were the first species with sequenced genome. *P*. *tricornutum* is a producer of the oxylipins 12-oxo-(5*Z*,8*Z*,10*E*)-dodecatrienoic acid and 9-oxo-(5*Z*,7*E*)-nonadienoic acid [27] and was reported to be affected by DD [8,28]. Exposure to this aldehyde altered the mitochondrial glutathione redox potential by oxidation of glutathione and induced cell death of *P*. *tricornutum* [28]. DD also triggers intracellular calcium transients and nitric oxide generation [8]. There is evidence for a sophisticated stress surveillance system in which individual diatom cells sense local DD concentration thereby monitoring the stress level of the entire population. An ortholog of the plant enzyme AtNOS1 was predicted as molecular target of PUAs [8]. Transcriptome analysis revealed that *PtNOA*, a gene with similarities to AtNOS1 [8], is upregulated in response to DD [29]. *PtNOA* overexpressing cell lines are hypersensitive to this PUA with altered expression of superoxide dismutase and metacaspases; both protein classes are involved in activation of programmed cell death [29]. Other studies on gene regulation in response to PUAs focused on copepods. In *Calanus helgolandicus* tubulin expression [30] and primary defense systems [31] were downregulated whereas detoxification genes like glutathione S-transferase, superoxide dismutase, and catalase remained unaffected [31] in response to a diet of the PUA producer *S*. *costatum* compared to a control.

**Fig 1:**
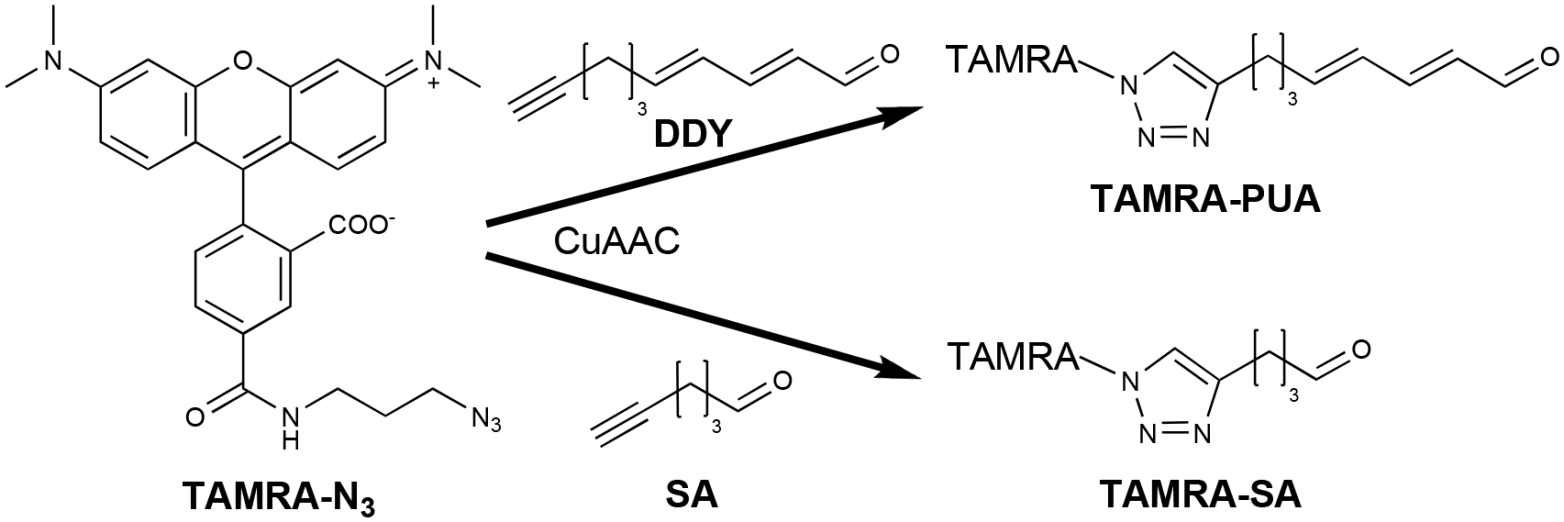
Synthesis of the probe TAMRA-PUA and the control TAMRA-SA. For details on the synthesis see [35].

We report here on the uptake, accumulation and molecular targets of a molecular probe containing a DD-derived head group and a 5-tetramethylrhodamine carboxamide fluorophore (TAMRA) reporter in *P*. *tricornutum* using an activity-based protein profiling (ABPP) strategy (Fig 1). Such chemical probe-enabled proteome strategies have been successfully applied with mechanism-based inhibitors [32] or protein-reactive natural products [33,34]. The utilized probe consists of a reactive group mimicking DD and a fluorescent reporter tag for detection [35]. By applying 2D gel electrophoresis (GE) followed by liquid chromatography/tandem mass spectrometry (LC-MS/MS) we found specific probe-labeled proteins having important roles regarding catalytic activity and biological functions in the alga including fucoxanthin chlorophyll *a*/*c* proteins, ATP synthases, a ribulose-phosphate-3-epimerase (RPE) and a phosphoribulokinase (PRK).

## Materials and Methods

### Uptake experiments

#### Growth of *P*. *tricornutum*

*P*. *tricornutum* (strain UTEX 646, Segelskär, Finland) was cultivated in artificial seawater prepared as described in Maier and Calenberg [36] under a 14/10 hours light/dark cycle, at 32 to 36 mmole photons s^-1^ m^-2^ and 13°C in 580 mL Weck jars (Weck, Wehr, Germany). The 4-(2-hydroxyethyl)-1-piperazineethanesulfonic acid (HEPES)-buffered medium was adjusted to a pH of 7.8 before autoclaving. Nutrient levels were 14.5 mM phosphate, 620 mM nitrate and 320 mM silicate. Incubation experiments were done under the same conditions.

#### Sample preparation

A *P*. *tricornutum* culture (100 μL) was pipetted on ethanol pre-cleaned cover slips (Marienfeld 474030-9010-000, 18x18 mm², D = 0.17 mm +/- 0.005 mm; Carl Zeiss Canada, Toronto, ON). To prevent evaporation cover slips were placed in a Petri dish, which contained a seawater wetted filter paper and were covered. Incubation for 8 hours allowed cells to adhere to the cover slips. Subsequently, 10μM of the substances DDY coupled to TAMRA (TAMRA-PUA), 5-hexynal (SA) coupled to TAMRA (TAMRA-SA) or azide modified TAMRA (TAMRA-N_3_) were added to the cell suspensions (each 0.5 mM stocks in DMSO), mixed gently with a pipette and incubated for one hour. Afterwards, the cell suspensions were removed and the cover slips were washed seven times by carefully pipetting 200 μL artificial seawater and incubated with 100 μL 4% [w/v] para-formaldehyde in artificial seawater for 25 min. The cover slips were washed twice with 200 μL artificial seawater and finally the liquid was removed. A control with the same washing and fixation steps was prepared as well. Due to extensive washing steps a part of algae cells detach from cover slips. 4 μL of 2,2’-thiodiethanol were pipetted on an ethanol pre-cleaned object slide and the treated side of the cover slip was placed on top of the object slide and slightly pressed down. Edges were fixed with nail polish and the slides were stored at 4°C until measurements on the next day. For each treatment three individual cover slips (biological replicates) were prepared out of three different *P*. *tricornutum* cultures. Those cultures have been prepared out of the same stock and kept under identical growth condition.

#### Fluorescence microscopy and analysis

*P*. *tricornutum* cells were observed with a structured illumination microscope (SIM), Zeiss Elyra S1 system (Carl Zeiss, Jena, Germany). Imaging was performed with an oil immersion objective lens (Plan-Apo, 63X, 1.4NA, Carl Zeiss, Jena, Germany). A 561 nm laser was used for excitation and fluorescence was filtered by a band pass filter (BP 570–620 nm) which opens up above 750 nm. 2D wide field images were acquired to compare fluorescence intensity of the different probes and treatments (TAMRA-PUA, TAMRA-SA, TAMRA-N_3_, no probe), whereas all cells were measured using the same settings (laser intensity, gain, exposure time). 12 cells were observed per treatment distributed over three biological replicates (microscope slides), four cells each. Three dimensional SIM images of treated *P*. *tricornutum* providing twofold resolution improvement were taken from selected samples to confirm, that TAMRA-PUA and TAMRA-SA were taken up in the cells and did not exclusively stick onto their surfaces. 15 raw images are required for reconstructing one 2D SIM image. 3D SIM images were taken with z steps of 110 nm. All SIM images were reconstructed on ZEN software 2010 (black edition, Carl Zeiss, Jena, Germany).

For fluorescence analysis bright field and wide field fluorescence images at 561 nm excitation were exported in TIF format with the ZEN software and processed with ImageJ2x 2.1.4.7 (freeware, http://www.rawak.de/ij2x/Download.html) as follows: tonal correction of the bright field image of each cell was optimized (see S1 Folder for unmodified images), the cell was encircled by hand and this selection was laid on the corresponding fluorescence picture using the ROI manager. The mean gray value, which is defined in this software as the sum of the gray values of all the pixels in the selection divided by the number of pixels, and the mean gray value of the background were measured and subtracted. For the previously described issue we use the term mean gray value per pixel. For background analysis a region of at least 1000 pixels was used.

### Probe labeling, 1D and 2D gel electrophoresis and identification of target proteins

#### *In vivo* labeling of *P*. *tricornutum* and sample preparation for gel electrophoresis

*P*. *tricornutum* cultures were grown for 13 days in 580 mL Weck jars (1.0 × 10^6^ to 1.5 × 10^6^ cells mL^-1^) without shaking as described before, which resulted in cells mainly sticking to the glass bottom. The overlaying artificial seawater was almost quantitatively removed with a pipette and cells were transferred into centrifuge tubes with the remaining medium.

For 1D GE samples were incubated with 100 μM DDY or 100 μM SA (each 50 mM stocks in DMSO) or DMSO for one hour.

For 2D GE the concentrated algae suspension was treated with 14 μL (250 μM) DDY stock solution (50 mM), which was added to 2.8 mL concentrated cell suspension (total cell number 48.7 × 10^6^ resulting in 14 fmol DDY cell^-1^) and incubated for 1 hour. During this incubation period no change in cell viability compared to untreated cells was observed by microscopy after application of Evan’s Blue [37]; however, we observed that incubation of several hours increases the amount of non-viable cells. The undiluted samples were centrifuged for 2 min at 1,800 g immediately after incubation to remove DDY and salts; the supernatant was removed and the cells were washed twice with 1 mL buffer A (10 mM HEPES and 250 mM sucrose, pH 7.4) and twice with 1 mL buffer B (10 mM HEPES and 250 mM sucrose, pH 8.2). After each washing step the tubes were centrifuged at 1,800 g for 2 min and the supernatant was discarded. Application of Evan’s Blue [37] and microscopy ensured that cells stayed intact during this procedure. The pellet was resuspended in buffer B and cells were treated with 1 mM dithiothreitol (20 mM stock in buffer B) to react with possibly unremoved DDY. Cells were lysed by sonication (ultrasonic probe: Bandelin sonotronic HD2070, power supply: Bandelin UW2070; Bandelin electronic, Berlin, Germany) twice for 15 s on ice.

The protein concentration was determined with the Roti^®^-Quant universal assay (Carl Roth, Karlsruhe, Germany) based on the biuret test using a microplate reader (Mithras LB 940, Berthold Technologies, Bad Wildbad, Germany) and bovine serum albumin as reference.

#### Copper(I)-catalyzed azide-alkyne cycloaddition (CuAAC)

*P*. *tricornutum* protein samples (30μL, 30 to 50 μg proteins μL^-1^) were diluted with 270 μL buffer B and incubated with 6 μL (0.09 mM) TAMRA-N_3_ solution (5 mM stock in DMSO), 18 μL (0.09 mM) ligand tris[(1-benzyl-1*H*-1,2,3-triazol-4-yl)methyl]amin solution (1.7 mM stock in DMSO/*tert*-butanol, 1/4, v/v) and 12 μL (35 mM) of a freshly prepared ascorbic acid solution (1.00 M in water). Samples were vortexed and 6 μL (0.88 mM) copper sulfate solution (50 mM in water) were added. Samples were vortexed again and stored on ice for 1 hour. 1% Triton™ X-100 and protease inhibitor cocktail (M221, Amresco Inc., Solon, OH, USA) were added according to the manufacturer’s protocol and after 30 min on ice samples were centrifuged at 15,000g and 3°C for 20 min. The supernatant was transferred into centrifugal filter units (vivaspin^®^500, 5,000 MWCO, PES, Sartorius, Göttingen, Germany) and the sample volume was reduced by centrifugation at 15,000g and 3°C. 100μL buffer B was added three times and the volume was reduced by centrifugation after each addition to give a final protein concentration of 10 to 20 μg proteins μL^-1^.

#### Sodium dodecyl sulfate polyacrylamide gel electrophoresis (SDS-PAGE) and in-gel fluorescence detection

20 μg of protein samples were mixed with 2× loading buffer [38] and heated to 95°C for 6 min. A protein ladder (PageRuler unstained protein ladder, Thermo Fischer Scientific Inc., Waltham, MA, USA) and the protein sample were loaded on a SDS mini gel containing 12% acrylamide resolving gel and 5% stacking gel prepared according to [39]. The gel was separated in a Mini-Protean^®^ Tetra gel cell (Bio-Rad, Herculas, CA, USA) by applying 80 V for 30min followed by 180 V for 65min. A fluorescent picture was taken at 312nm irradiation using a UV transilluminator (UV star, Bio-Rad), a PowerShot A640 camera (Canon, Tokyo, Japan) and a 520 nm long pass filter. The gel was stained with RAPIDstain™ (G-Biosciences, St. Louis, MO, USA).

#### Difference gel electrophoresis (DIGE)

DIGE was conducted in triplicates. 440 to 880 μg protein of the probe-treated sample (incubated with DDY and connected with TAMRA-N_3_ by CuAAC as described before) and 50 μg protein of a control sample incubated with the N-hydroxysuccinimide ester of the cyanine 5 fluorophore (Cy5 NHS ester) were loaded on each of the isoelectric focusing (IEF) strips (Immobiline DryStrip, 24 cm, pH 3–11 NL, GE Healthcare Bio- Sciences, Piscataway, NJ, USA). For Cy5 labeling of the whole proteome 20 μL of the control sample were diluted with 48 μL buffer B. 3 μL Cy5 NHS ester (2 mM in DMSO) were added and the mixture was incubated with shaking at 4°C for 45 min. To stop labeling 5 μL of a 10 mM solution of L-lysine in water were added.

The IEF strip was rehydrated overnight (7 M urea, 2 M thiourea, 2% [w/v] CHAPS, 0.002% [w/v] bromophenol blue, 0.5% [v/v] IPG buffer (GE Healthcare Bio-Sciences), 10 mM dithiothreitol). Isoelectric focusing of the strips with the Ettan IPGphor II (GE Healthcare Bio-Sciences) was carried out according to the following protocol: 4 h at 300 V (gradient), 4 h at 600 V (gradient), 4 h at 1,000 V (gradient), 4 h at 8,000 V (gradient) and 3 h at 8,000 V (step).

After isoelectric focusing the IEF strips were equilibrated for 15 min in 10 mL of equilibration buffer (6 M urea, 30% [v/v] glycerol, 2% [w/v] SDS, 75 mM tris(hydroxymethyl)aminomethane, 0.002% [w/v] bromophenol blue) containing 1% [w/v] dithiothreitol and subsequently for 15 min in 10 mL of equilibration buffer containing 2.5% [w/v] iodoacetamide. For separation of proteins in the second dimension, the Ettan DALT System (GE Healthcare Bio-Sciences) was used. SDS polyacrylamide gels 12% [w/v] of 1.0 mm thickness were casted via the Ettan DALTsix Gel caster (GE Healthcare Bio-Sciences). The separation conditions were as follows: 1 W/gel for 1 h followed by 15 W/gel for 5 h. Proteins were visualized by analyzing the gels with a Typhoon 9410 scanner (GE Healthcare Bio-Sciences) using a resolution of 100 μm. Proteins were fixed (10% [v/v] acetic acid, 50% [v,v] methanol in water), stained (0,025% [w/v] Coomassie R 250, 10% [v/v] acetic acid in water) and the gels were destained (10% [v/v] acetic acid in water; see S3 Folder for unmodified Coomassie and fluorescence images of the gels). Gels were merged with Delta2D (DECODON, Greifswald, Germany).

#### Protein isolation, LC-MS/MS analysis and data processing

Fluorescent TAMRA-PUA protein spots were in-gel reduced, alkylated with iodoacetamide and digested as described by Shevchenko et al. [40]. Tryptic peptides were extracted, dried down in a vacuum centrifuge and dissolved in 10 μL of water containing 0.1% formic acid.

LC-MS/MS analysis and data processing were conducted as described in S1 Information.

## Results

### Probe design and labeling strategy

**Fig 2:**
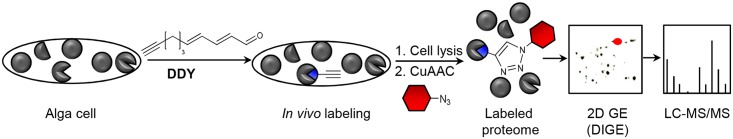
Schematic *in vivo* application of the probe. Living cells of *P*. *tricornutum* were incubated with the PUA-derivative DDY. After removal of excess DDY cell lysis followed by CuAAC enables attachment of the fluorescent reporter to covalently labeled proteins. 1D GE quickly allows detection of labeled proteins (not shown). Identification of protein targets is enabled by 2D GE. Therefore, a second *P*. *tricornutum* sample was treated with Cy5 NHS ester to label the whole proteome. The combined samples were separated using DIGE, labeled proteins were digested using trypsin and the resulting peptides were separated and analyzed by LC-MS/MS.

The fluorescent α,β,γ,δ-unsaturated aldehyde-derived probe TAMRA-PUA (Fig 1) could be used successfully to investigate uptake and accumulation of PUAs in *P*. *tricornutum* and to monitor protein targets of these natural products. TAMRA-PUA was recently developed in our group to monitor accumulation of PUAs in copepods [35]. The probe consists of DDY as reactive group that mimics DD. The alkyne functionality allows to couple the commercially available azide modified tetramethylrhodamine TAMRA-N_3_ (Fig 1). To identify protein targets, DDY was incubated with *P*. *tricornutum* cells. After work-up CuAAC allowed to covalently link the reporter TAMRA-N_3_ to DDY (Figs 1 and 2). For uptake studies we employed the probe as already coupled construct TAMRA-PUA. For comparison of the activity of α,β,γ,δ-unsaturated aldehyde-derived probes with structurally related saturated-aldehyde-derived ones we also applied the probe TAMRA-SA (Fig 1).

### TAMRA-PUA accumulates in *P*. *tricornutum*

**Fig 3:**
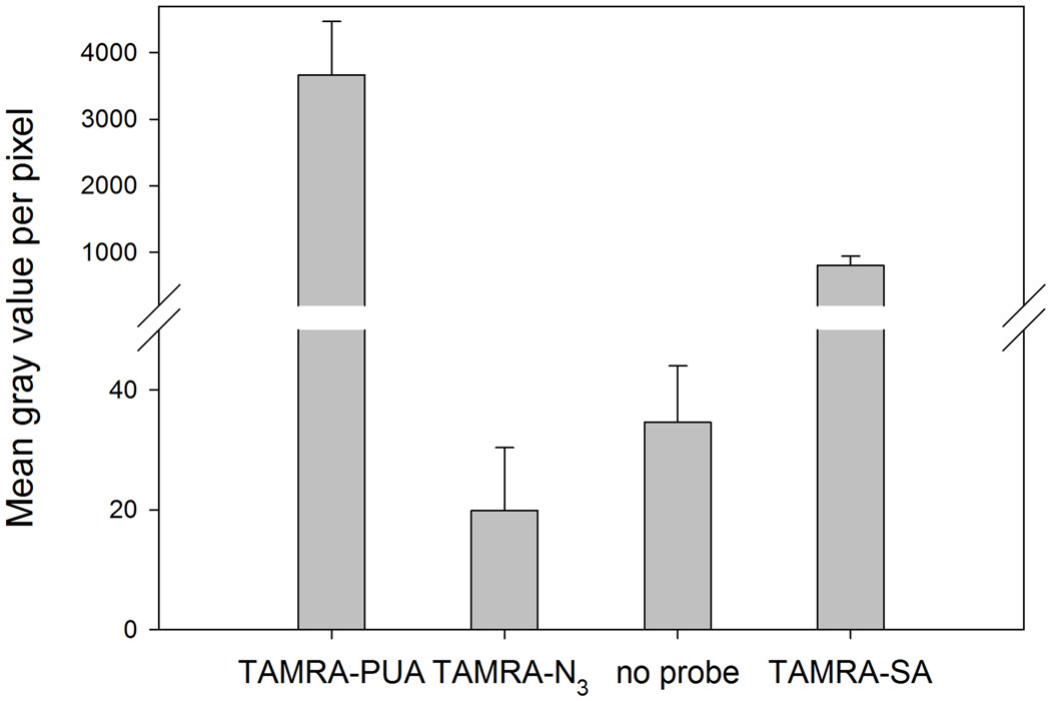
Fluorescence intensity of *P*. *tricornutum* cells treated under different conditions. Cells were either incubated with TAMRA-PUA, TAMRA-N_3_, TAMRA-SA for one hour or kept under identical conditions without probe. For each treatment three microscope slides with four cells each were measured. Unmodified raw data are available in S1 and S2 Folders. Fluorescence intensities were recorded as mean gray value per pixel after data treatment as described in the main text. Averaged mean gray values per pixel of cells of each treatment are presented as bars ±SD. One way Anova comparing results of different microscope slides within one treatment revealed no statistical difference (p>0.05). Kruskal-Wallis one way analysis of variance on ranks revealed differences in the median values among the treatment groups (H = 42.436, p<0.001) and Tukey’s HSD test (p<0.05) allowed classification into three groups: (a) TAMRA-PUA with the highest mean gray value per pixel of 3661±809, (b) TAMRA-SA with 800±140 and (c) TAMRA-N_3_ and control with almost no emission signals (20±10 and 35±9); these controls were not significantly different to each other (Tukey’s HSD test p>0.05).

Cell permeability and uptake of the probes by *P*. *tricornutum* was investigated by wide field fluorescence microscopy. Living cells were treated with TAMRA-PUA, TAMRA-SA or TAMRA-N_3_ for labeling or kept as control without additional treatment. After one hour incubation probes were removed by washing seven times with artificial seawater, once with artificial seawater containing 4% paraformaldehyde for cell fixation and twice with artificial seawater. Cells were embedded in 2,2’-thiodiethanol and measured with an epifluorescence microscope in wide field mode. Images were processed with ImageJ. Cells were encircled by hand and the background corrected average mean gray value per pixel within each alga cell was calculated (Fig 3). The aldehyde containing probes TAMRA-PUA and TAMRA-SA were significantly enriched in the cells compared to TAMRA-N_3_ or the control. Interestingly, TAMRA-PUA accumulation was significantly higher compared to TAMRA-SA, despite being similar in physicochemical properties.

**Fig 4:**
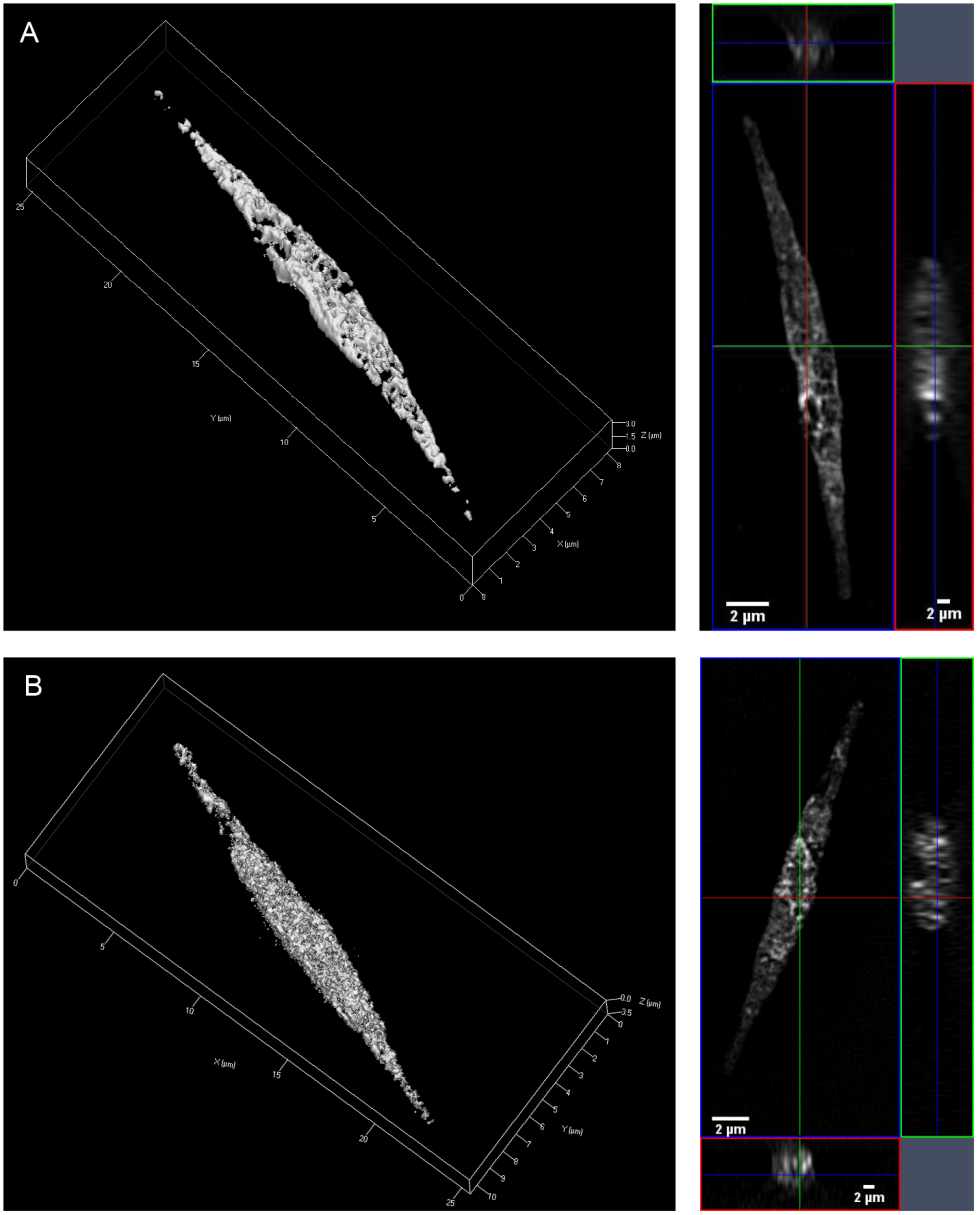
Fluorescence microscopy of *P*. *tricornutum* cells. 3D (left) and 2D (right) images of a TAMRA-PUA (A) and a TAMRA-SA (B) treated cell. Images were taken in 3D SIM mode. A 561nm laser was used for excitation, and fluorescence was filtered by a band pass filter (BP 570-620nm) which opens up above 750nm. Fluorescence is visible in the entire cells, which confirms that both probes were taken up.

Results were verified in additional independent experiments, also using a different embedding medium (S1 Fig). To confirm that the probes do not only appear on the surface we conducted 3D SIM showing that TAMRA-PUA and TAMRA-SA accumulate within the cells (Fig 4). Distribution of label revealed local maxima but in general nearly the entire cellular content was affected by the probe.

### DDY covalently modifies proteins of *P*. *tricornutum*

We next tested for protein targets of PUAs in living cells using DDY as well as the saturated aldehyde derivative SA. After incubation with the probes followed by cell lysis, TAMRA-N_3_ was coupled to the alkyne groups of DDY as well as SA via CuAAC (Fig 2). Proteins were separated by 1D GE and monitored for fluorescent labeling. UV-illumination revealed exclusive labeling of proteins in DDY treated cells while SA and DMSO treatments did not result in any fluorescent bands (S2 Fig).

To unravel protein targets of DDY, protein extracts obtained after incubation of *P*. *tricornutum* with the probe as described above were separated by DIGE in three replicates (S3 Fig, S3 Folder for unmodified pictures). For comparison samples without probe addition were incubated with the Cy5 NHS ester to label the whole proteome. Separation was performed by isoelectric focusing and SDS-PAGE as second dimension and DDY-TAMRA labeled proteins were excised and tryptically digested. Digested peptides were separated and analyzed by LC-MS/MS (Fig 2). More precisely separation was conducted with a nano Ultra Performance Liquid Chromatography (nanoUPLC) and analysis by tandem mass spectrometry using data-independent acquisition (MS^E^ analysis). In data-independent analysis the mass spectrometer cycles between low and high energy acquisition of data resulting in high sampling rate.

A list of confident target proteins classified according to their biological processes and molecular functions is shown in Table 1. Two ATP synthases, four different chlorophyll *a*/*c* binding proteins, different catalytic active enzymes and some predicted proteins were found to be modified by the probe. A full list of confident, probable and putative proteins (for evaluation see S1 Information) as well as an overview of all protein hits for each gel is given in S1 Table.

**Table 1:** Confident target proteins found by 2D GE. ^1^

Protein	Gene name	Accession No.	Mass (kDa)	Gel 1	Gel 2	Gel 3
**1) Biological process**						
**ATP biosynthetic process**						
ATP synthase subunit alpha, chloroplastic	*AtpA*	A0T0F1	54621.6	X	OO	O
ATP synthase subunit beta	*AtpB*	B7FS46	53619.2	X	OO	O
**Photosynthesis**						
Fucoxanthin chlorophyll a/c protein	*Lhcf10*	B7G5B6	21352.7	X	OOO	O
Fucoxanthin chlorophyll a/c protein	*Lhcf9*	B7G955	22100.5	OOO	O	X
Fucoxanthin chlorophyll a/c protein	*Lhcx2*	B7FR60	21177.4	OO	O	X
Fucoxanthin chlorophyll a/c protein	*Lhcf4*	B7FRW2	21328.5	OO	O	X
**2) Molecular function**						
**Catalytic activity, isomerase activity**						
Ribulose-phosphate 3-epimerase	*Rpe*	B7FRG3	27812.0	OO	O	O
**Catalytic activity, ligase activity**						
Predicted protein, family: Aspartate-ammonia ligase	*PHATRDRAFT_44902* ^*a*^	B7FW24	43206.1	X	OO	O
**Catalytic activity, oxidoreductase activity**						
Predicted protein, domains: Thioredoxin-like fold, Thioredoxin domain	*PHATRDRAFT_42566* ^*a*^	B7FNS4	24136.3	X	OOO	OOO
Predicted protein, domain: Rieske [2Fe-2S] iron-sulphur domain	*PHATRDRAFT_9046* ^*a*^	B7FPI8	17010.4	X	OO	O
**Catalytic activity, transferase activity**						
Phosphoribulokinase	*Prk*	B5Y5F0	43325.4	X	OO	O
**3) Predicted proteins without assignable function**						
Predicted protein	*PHATRDRAFT_42612* ^*a*^	B7FNX7	24938.5	O	OOO	OOO
Predicted protein	*PHATRDRAFT_45465* ^*a*^ */ PHATRDRAFT_50215* ^*a*^	B7FXS8	37645.2	O	X	OOO
Predicted protein, family: SOUL haem-binding protein	*PHATRDRAFT_37136* ^*a*^	B7G284	46049.4	O	OOO	OOO
Predicted protein	*PHATRDRAFT_49286* ^*a*^	B7GA37	32141.6	OO	O	O
Predicted protein, domain: NAD(P)-binding domain	*PHATRDRAFT_49287* ^*a*^	B7GA38	126885.6	X	OO	O
Predicted protein, family: Protein of unknown function DUF1517	*PHATRDRAFT_32071* ^*a*^	B7FQ47	33258.8	OO	X	O

^1^Proteins in this table were found in at least two of the three gels, a full list of labeled proteins can be found in S1 Information. OOO—only one protein per excised gel spot was found, OO—identification of probe labeled protein besides other unlabeled proteins in a gel spot, O—more than one labeled protein per excised gel spot, X—no hit.

^a^Names are temporarily ascribed to an open reading frame (ORF) by a sequencing project [41].

## Discussion

While previous research mainly has reported the teratogenic and allelochemical effects of PUAs as well as their role in cell to cell signaling (reviewed in [2–5]) and PUAs influence on gene regulation [30,31], almost nothing is known about underlying mechanistic aspects regarding covalent protein interactions. Therefore, we applied a PUA-derived as well as control probe to *P*. *tricornutum*.

### Probe design and labeling strategy

PUAs are known to have diverse effects on planktonic organisms but defined molecular targets are hitherto almost unidentified. Especially their function in cell to cell communication of diatoms has attracted much attention [7,8,10,14]. We undertook a labeling study to obtain a deeper insight into the mechanism of action of these metabolites to reveal potential PUA-uptake of phytoplankton and to identify protein targets of the compounds. Following previous structure activity studies we addressed the specific activity of PUAs by comparison of probes derived from the active unsaturated aldehyde (TAMRA-PUA) and the inactive saturated aldehyde (TAMRA-SA) [16,35]. The design of the probes allowed to employ them in two different modes. For uptake studies we could use the TAMRA coupled molecules as described earlier for the monitoring of PUA-targeting in copepods [35]. Interestingly, the different effects of unmodified saturated aldehydes and PUAs observed in previous studies [16] were also mirrored in the effect of our different TAMRA-constructs. This indicates that the TAMRA substitution has no significant influence on the action of the aldehydes. However we cannot exclude that permeability is altered by the substitution.

Probe concentration was set to 10μM, a value for which different algae showed response to DD regarding cell membrane permeability of SYTOX Green [7], but *P*. *tricornutum* did not [8]. For identification of protein targets we developed a two-step protocol involving incubation with unmodified SA or DDY and, after work-up, coupling with the TAMRA-N_3_. This approach allows to minimize the influence of bulky groups during *in vivo* interaction with target proteins. The well-established CuAAC coupling allowed to covalently link the dye to DDY-labeled proteins [42,43]. As fluorescent reporter we selected the tetramethylrhodamine fluorophore as it is relatively cheap, pH insensitive, photostable, cell permeable and easily excitable with common lasers and filter sets [44]. Compared to experimental design of fluorescence microscopy where physiological conclusions were relevant, probe concentration in the mechanistic gel electrophoretic experiments was increased to 250μM DDY ensuring an adequate detection of labeled proteins.

### TAMRA-PUA accumulates in *P*. *tricornutum*

Fluorescence microscopy clearly shows an uptake and accumulation of the DD derived probe by *P*. *tricornutum* (Fig 3). In contrast, TAMRA-SA, the probe derived from an almost inactive aldehyde with otherwise similar physicochemical properties, compared to TAMRA-PUA, did not substantially accumulate in the cells. Apparently the α,β,γ,δ-unsaturated structure element found in PUAs is responsible for uptake and/or accumulation within diatom cells. A potential mechanism explaining the accumulation could be an inhibited exfiltration due to covalent adduct formation with cellular components such as proteins [17,18] as verified below or DNA [19–21]. In contrast, the weaker fluorescence signal of TAMRA-SA is consistent with the much lower reactivity of the underlying structure hexanal for which only few covalent reactions with proteins have been reported [45,46]. By applying the hexanal derived TAMRA-SA to a *P*. *tricornutum* culture we did not observe any covalently modified proteins in the corresponding 1D gel (S2 Fig).

The intracellular accumulation can explain the specific elicitation of effects by PUAs [8]. We can exclude that the dye itself accumulates unspecifically in cells since TAMRA-N_3_ treated *P*. *tricornutum* showed no different fluorescence signals compared to untreated controls (Tukey’s HSD test p>0.05 between TAMRA-N_3_ and no probe), confirming the effective washing procedure to remove TAMRA [47]. 3D SIM images (Fig 4) reveal that fluorescence after application of the TAMRA-PUA probe is distributed over almost the entire cell. The lack of intracellular compartmentation can be explained with the high reactivity of such types of electrophilic Michael acceptors [16,48,49]. Apparently no preferred shuttling of the probe into specific compartments occurs but also the cell walls and membranes do not represent a barrier for this compound class. PUA-uptake might thus be a way to facilitate diatoms´ perception of this compound class. Efficient uptake of essential metabolites has been earlier observed in diatoms but specific uptake mechanisms of primary and secondary metabolites involving transporters are not yet identified [50]. However, transporters of glucose that can support mixotrophic growth of *P*. *tricornutum* are known, supporting the note of the capability of the alga to actively take up organic metabolites from its environment [51]. This further supports the notion of a possible cell to cell communication mechanism based on PUAs that requires cellular uptake. To unravel potential molecular targets within the proteome of *P*. *tricornutum* we undertook further labeling studies.

### DDY covalently modifies proteins of *P*. *tricornutum*

We performed an *in vivo* labeling of *P*. *tricornutum* with a PUA-derived probe to identify target proteins and to deduce affected molecular functions and biochemical pathways. We hypothesize that modified proteins may lose their function and that PUAs thereby interrupt or disturb metabolic pathways. These changes on a molecular level probably lead to observed effects of PUAs like growth inhibition and cell death [4,8].

Whereas Vardi et al. used a transcriptome analysis to search for DD affected genes and gene products by screening for up- and downregulated transcripts [29], we performed a direct investigation on the covalent modification of the proteome by DDY. Thus, we discover interactions with proteins, which do not necessarily have an influence on the transcript level but a direct influence on the functionality of these proteins.

Although the unsaturated aldehyde group of PUAs is universally reactive against nucleophilic amino acid side chains, we received moderate specific labeling of proteins (Table 1). This agrees with previous findings that DD preferentially attacks distinct proteins and specific nucleophilic sites if incubated with isolated purified proteins [17]. Underneath the confident target proteins we found four fucoxanthin chlorophyll *a*/*c* proteins, which are part of the light harvesting complex (LHC), responsible for the delivery of excitation energy between photosystem I and II [52]. Compared to higher plants, LHCs of diatoms named fucoxanthin-chlorophyll-proteins bind chlorophyll *c* instead of *b* and fucoxanthin instead of lutein [53]. Three groups of LHCs regarding their sequence and function are known, the found target proteins (see Table 1) belong to two groups of them: *Lhcx* gene products are needed for protection against surplus light and thus photoprotection and *Lhcf* gene products, the main antenna proteins, function in light harvesting (reviewed in [54]). Effects of DD on photosystem efficiency have already been shown for the diatoms *Thalassiosira weissflogii* [14] and a transgenic *P*. *tricornutum* [29] and our findings now provide a mechanistic explanation for this action of PUAs.

The energy harvested during light reaction is mainly stored by forming adenosine triphosphate (ATP). We identified two probe labeled ATP synthase subunits (see Table 1) belonging to the extrinsic catalytic sector, CF1 [55] of the chloroplastic ATP synthase. ATP synthase, located either in the mitochondria inner membrane or chloroplast thylakoid membrane, are responsible for cellular ATP production from adenosine diphosphate and inorganic phosphor in the presence of a proton gradient across the membrane [56]. The two PUA-targets ATP synthase subunit alpha (*AtpA*) and ATP synthase subunit beta (A*tpB*) are located in the water soluble CF1 complex of the chloroplastic ATP synthase. In the green algae *Chlamydomonas reinhardtii* in the absence of the mitochondrial beta-subunit (gene name *Atp2*), ATP synthase could not be assembled into an enzyme complex leading to decreased mitochondrial respiration [57]. In conjunction with the finding of impairment of enzymes involved in light harvesting the identified ATP synthase targets support the notion of a profound modulation of the energy household under the influence of PUAs.

Enzymes from the Calvin cycle are also PUA-targets connected to photosynthetic activity. Two PUA-targets, the kinase PRK and the epimerase RPE were found to be labeled after DDY incubation. These enzymes are involved in carbon dioxide assimilation during the dark reaction. RPE reversibly catalyzes the reaction of D-xylulose 5-phosphate to D-ribulose 5-phosphate in the Calvin cycle and pentose phosphate pathway [58]. The product D-ribulose-5-phosphate is under ATP consumption further converted by PRK to D-ribulose-1,5-bisphosphate, which acts as acceptor for CO_2_ in photosynthetic carbon assimilation [59]. In the Calvin cycle glyceraldehyde-3-phosphate dehydrogenase, the small protein CP12 and PRK form a multi-enzyme complex, the redox state of PRK is regulated by thioredoxin-mediated thiol-disulfide exchange in a light-dependent manner [59,60]. PRK is not active in the oxidized form where cysteine residues at positions 16 and 55 in land plants and green algae form an intramolecular disulfide bridge [61]. By reaction of those thiols with a PUA inactivation of PRK due to spatial changes and loss of redox behavior is conceivable. Also labeling on other sites, such as Lys may lead to loss of activity. Examples for alkylations were shown for PRK of different origin [62,63] and accordingly, alkylation of thiols and other nucleophilic residues by PUAs might change activity of enzymes.

It is striking that metabolic pathways such as the pentose phosphate pathway, photosynthesis including photophosphorylation and Calvin cycle that are involved in the energy household are specifically affected by PUA-treatment. The response is more immediate than transcriptomic regulation since proteins are the direct target of a covalent modification.

## Conclusion

In this study we investigate the structure specificity of the uptake of PUA-derived probes and analogues in *P*. *tricornutum*. We could also reveal PUA probe targets within the proteome of the alga. Uptake experiments show a clear enrichment of TAMRA-PUA within the cells compared to TAMRA-SA. Chemoproteomics allowed the identification of target proteins of TAMRA-PUA. Interestingly, preferential targets have important roles in biological processes covering photosynthesis including ATP generation, conversion in Calvin cycle or the pentose phosphate pathway. Besides three *Lhcf*- and one *Lhcx*-coding proteins important for light harvesting and photoprotection we found two ATP synthases. Generation of ATP is of major importance since it supports nearly all cellular activities that require energy and its synthesis is the most frequent chemical reaction in the biological word [56]. PRK, another PUA target catalyzes the only reaction by which intermediates in the Calvin cycle can be contributed for further CO_2_ assimilation [64]. RPE is important for both, the Calvin cycle and the reverse pentose phosphate pathway. Loss of molecular functions of these proteins as it might occur through covalent reactions of the nucleophilic protein residues with a PUA would immediately interfere with the homeostasis of algae cells, explaining the fast adverse effect of PUAs.

## Supporting Information

S1 Fig Relative fluorescence intensity of *P*. *tricornutum* cells treated under different conditions in two independent experiments. Cells were either incubated with TAMRA-PUA, TAMRA-N_3_, TAMRA-SA (only experiment 2) for one hour or kept under identical conditions without probe. For each experiment one microscope slide per treatment with five cells (experiment 1) or seven cells (experiment 2) was measured. For experiment 1, all microscope slides were embedded in 2,2’-thiodiethanol as described in the materials and methods section, for experiment 2 a poly (vinyl alcohol)/ n-propyl gallate antifade embedding medium [See Lu-Walther H-W, Kielhorn M, Förster R, Jost A, Wicker K, Heintzmann R. fastSIM: a practical implementation of fast structured illumination microscopy. Methods Appl Fluoresc. 2015;3(1):014001] was used. Fluorescence intensities were recorded as mean gray value per pixel after data treatment as described in the main text. To compare both experiments results were normalized to “no probe”. Normalized averaged mean gray values per pixel of cells of each treatment are presented as bars ±SD. Kruskal-Wallis one way analysis of variance on ranks revealed differences in the median values among the treatment groups of each experiment (No. 1 H = 12.500, No. 2 H = 22.902; p<0.05). Tukey’s HSD test (p<0.05) attested significant differences between TAMRA-PUA and all other treatments within each experiment.
(TIF)

S2 Fig SDS-PAGE of *in vivo* treated samples of *P*. *tricornutum*. *P*. *tricornutum* was incubated with 100μM of the reactive group (RG) DDY or SA or DMSO as control. After one hour incubation cells were lysed, CuAAC with TAMRA-N_**3**_ was applied and SDS-PAGE and in-gel fluorescence detection were accomplished (see also Fig 2). Only the DDY treated sample shows specific fluorescent bands.
(TIF)

S3 Fig 2D GE images. Position of excised spots with identified proteins in the three 2D gels (1, 2 and 3) presented in the Coomassie stained gels (A) and fluorescence images excited at 532nm for TAMRA-PUA detection (B). The positions of the spots were computed by Delta 2D for each image by considering the Coomassie stained gel image as well as TAMRA-PUA and Cy5 fluorescence images (for raw data of each image see S3 Folder). Slightly shifted positions of spots between Coomassie and fluorescence images of each gel are due to change of gel dimensions during Coomassie staining.
(TIF)

S1 Folder Unmodified wide field fluorescence images of *P*. *tricornutum* treated with TAMRA-PUA, TAMRA-SA, TAMRA-N_3_ or without addition of a substance as control for uptake experiments. Cells were measured with a Zeiss Elyra S1 system in wide field mode. A 561 nm laser was used for excitation, and fluorescence was filtered by a band pass filter (BP 570–620 nm) which opens up above 750 nm. WF—wide field.
(ZIP)

S2 Folder Unmodified bright field images of *P*. *tricornutum* treated with TAMRA-PUA, TAMRA-SA, TAMRA-N_3_ or without addition of a substance as control for uptake experiments. Cells were measured with a Zeiss Elyra S1 system in bright field mode. Before data analysis tonal correction was optimized. BF—bright field.
(ZIP)

S3 Folder Unmodified 2D GE images. Fluorescence images of TAMRA-PUA and Cy5 labeled protein gels and images of Coomassie stained gels.
(ZIP)

S1 Information LC-MS/MS analysis and data processing. (DOCX)

S1 Table Target proteins found by 2D GE. Proteins were classified into confident, labeled and putative proteins subject to guidelines described in S1 Information and separated according to their biological processes and molecular functions.
(XLSX)
