# Supplementary figures and images for: A Metabolic Probe-Enabled Strategy Reveals Uptake and Protein Targets of Polyunsaturated Aldehydes in the Diatom Phaeodactylum tricornutum
Source: PLoS One. 2015 Oct 23;10(10):e0140927. doi: 10.1371/journal.pone.0140927 (PMC4619725; doi:10.1371/journal.pone.0140927)

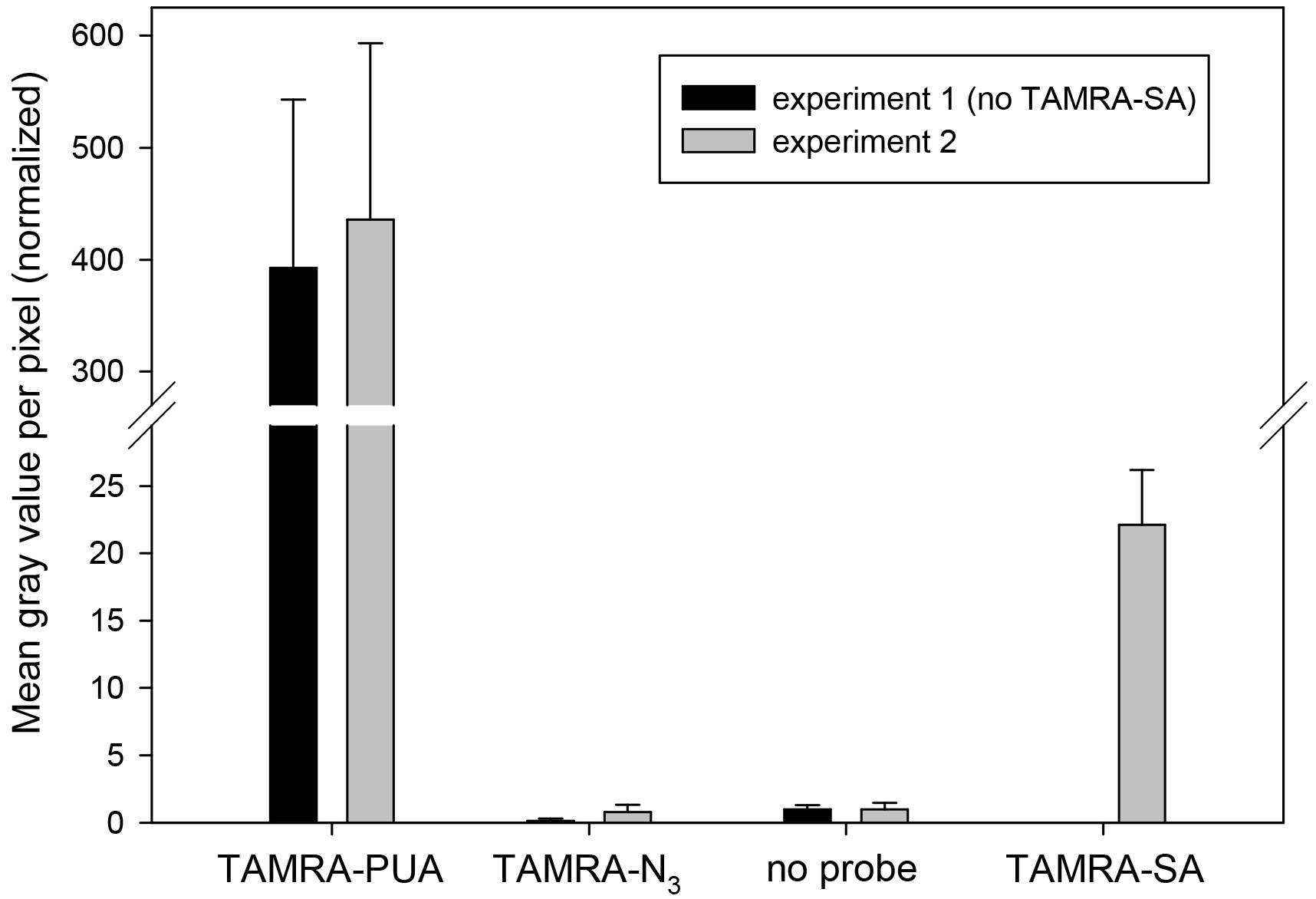

Supplement: S1 Fig — Cells were either incubated with TAMRA-PUA, TAMRA-N3, TAMRA-SA (only experiment 2) for one hour or kept under identical conditions without probe. For each experiment one microscope slide per treatment with five cells (experiment 1) or seven cells (experiment 2) was measured. For experiment 1, all microscope slides were embedded in 2,2’-thiodiethanol as described in the materials and methods section, for experiment 2 a poly (vinyl alcohol)/ n-propyl gallate antifade embedding medium [See Lu-Walther H-W, Kielhorn M, Förster R, Jost A, Wicker K, Heintzmann R. fastSIM: a practical implementation of fast structured illumination microscopy. Methods Appl Fluoresc. 2015;3(1):014001] was used. Fluorescence intensities were recorded as mean gray value per pixel after data treatment as described in the main text. To compare both experiments results were normalized to “no probe”. Normalized averaged mean gray values per pixel of cells of each treatment are presented as bars ±SD. Kruskal-Wallis one way analysis of variance on ranks revealed differences in the median values among the treatment groups of each experiment (No. 1 H = 12.500, No. 2 H = 22.902; p<0.05). Tukey’s HSD test (p<0.05) attested significant differences between TAMRA-PUA and all other treatments within each experiment. (TIF) [file pone.0140927.s001.tif]

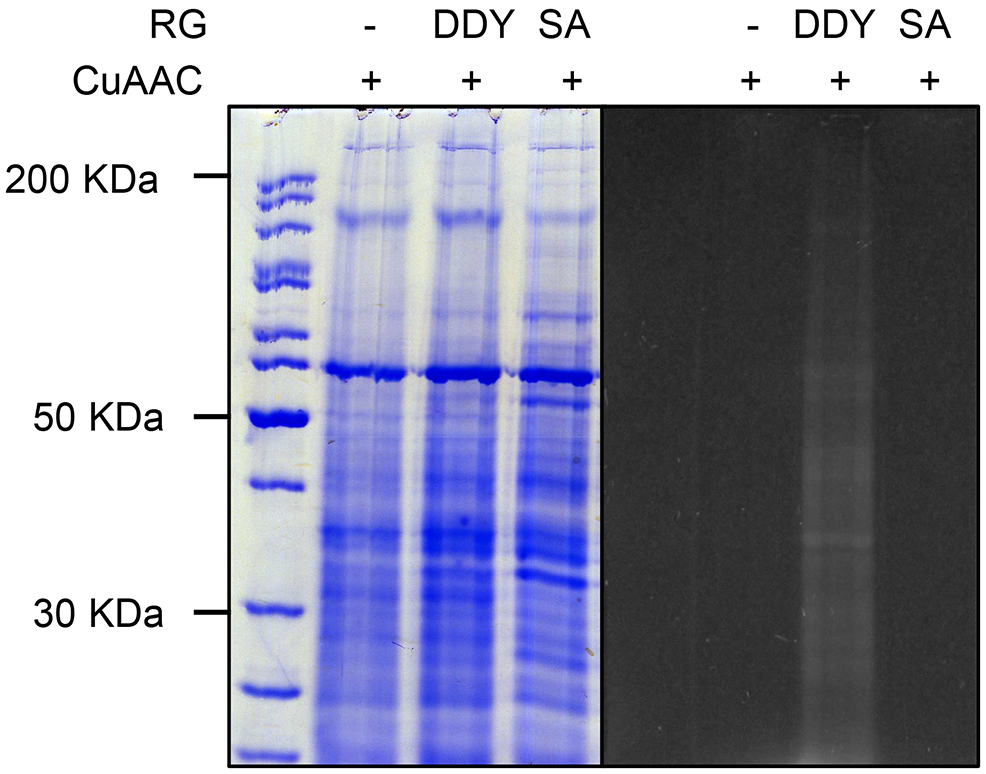

Supplement: S2 Fig — P. tricornutum was incubated with 100μM of the reactive group (RG) DDY or SA or DMSO as control. After one hour incubation cells were lysed, CuAAC with TAMRA-N3 was applied and SDS-PAGE and in-gel fluorescence detection were accomplished (see also Fig 2). Only the DDY treated sample shows specific fluorescent bands. (TIF) [file pone.0140927.s002.tif]

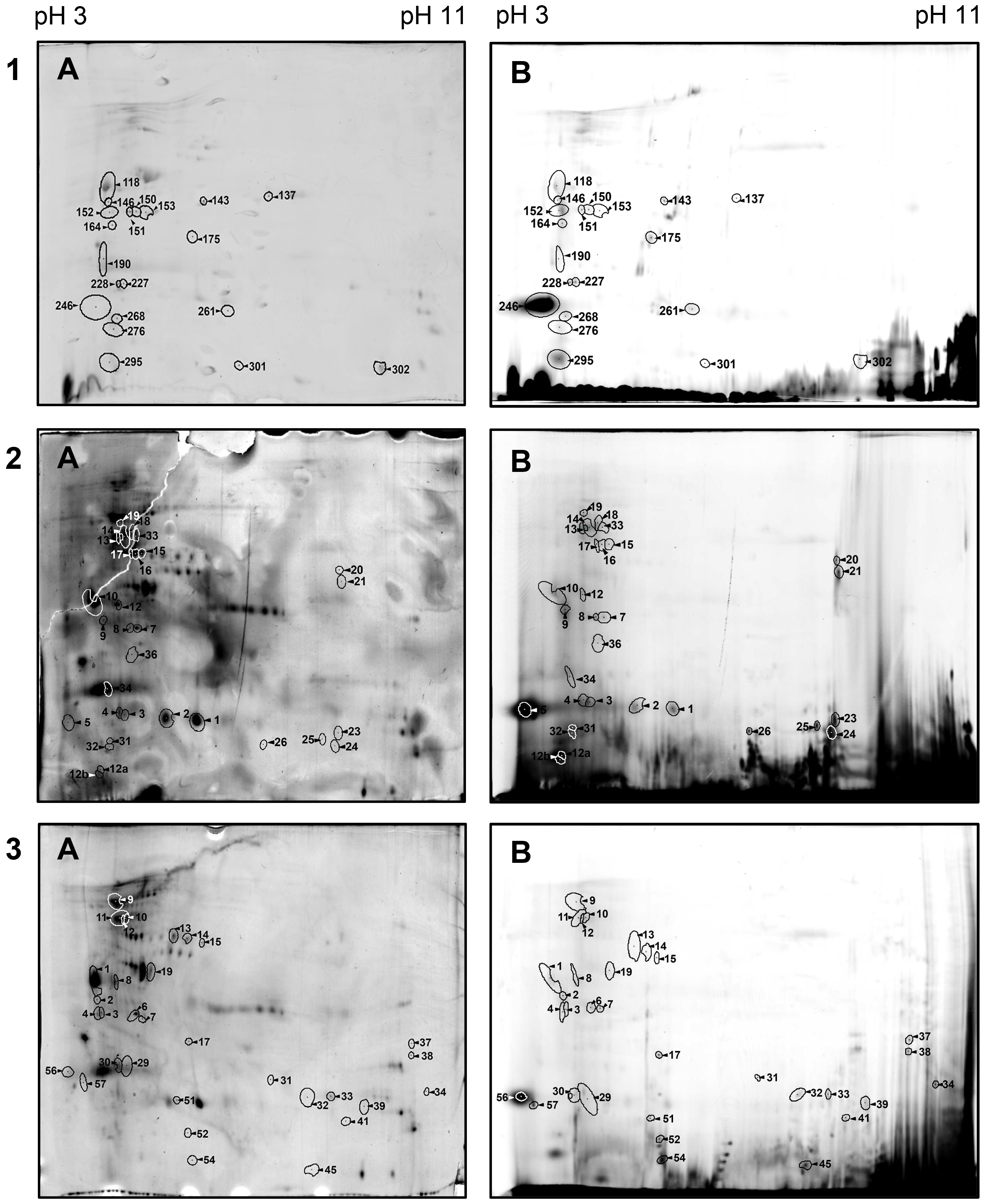

Supplement: S3 Fig — Position of excised spots with identified proteins in the three 2D gels (1, 2 and 3) presented in the Coomassie stained gels (A) and fluorescence images excited at 532nm for TAMRA-PUA detection (B). The positions of the spots were computed by Delta 2D for each image by considering the Coomassie stained gel image as well as TAMRA-PUA and Cy5 fluorescence images (for raw data of each image see S3 Folder). Slightly shifted positions of spots between Coomassie and fluorescence images of each gel are due to change of gel dimensions during Coomassie staining. (TIF) [file pone.0140927.s003.tif]
