# Supplementary material for: A Metabolic Probe-Enabled Strategy Reveals Uptake and Protein Targets of Polyunsaturated Aldehydes in the Diatom Phaeodactylum tricornutum
Source: PLoS One. 2015 Oct 23;10(10):e0140927. doi: 10.1371/journal.pone.0140927 (PMC4619725; doi:10.1371/journal.pone.0140927)
Supplement: S1 Information — (DOCX) [file pone.0140927.s007.docx]

# S1 Supporting Information. LC-MS/MS analysis and data processing

A metabolic probe-enabled strategy reveals uptake and protein targets of polyunsaturated aldehydes in the diatom *Phaeodactylum tricornutum*

Stefanie Wolfram^1^, Natalie Wielsch^2^, Yvonne Hupfer^2^, Bettina Mönch^3^, Hui-Wen Lu-Walther^4^, Rainer Heintzmann^4,5^, Oliver Werz^3^, Aleš Svatoš^2^, Georg Pohnert^1^*

^1^Bioorganic Analytics, Institute for Inorganic and Analytical Chemistry, Friedrich Schiller University, Jena, Germany

^2^Department Mass Spectrometry/Proteomics, Max Planck Institute for Chemical Ecology, Jena, Germany

^3^Department of Pharmaceutical and Medicinal Chemistry, Institute of Pharmacy, Friedrich Schiller University, Jena, Germany

^4^Biomedical Imaging, Department Microscopy, Leibniz Institute of Photonic Technology e.V., Jena, Germany

^5^Institute for Physical Chemistry, Abbe Center of Photonics, Friedrich Schiller University, Jena, Germany

* Corresponding author

E-mail: Georg.Pohnert@uni-jena.de

**LC-MS/MS analysis**

After protein reduction, alkylation and digestion, 1 to 8 µL of the peptide mixture depending on staining intensity were injected onto a nanoAcquity nanoUPLC system (Waters, Milford, MA, USA) online coupled to a Q-ToF HDMS mass spectrometer (Waters). Peptides were desalted using a Symmetry C18 trap-column (20 x 0.18 mm, 5 µm particle size) at a flow rate of 15 µL min^-1^ (0.1% aqueous formic acid (FA)) and then eluted onto a nanoAcquity C18 analytical column (200 mm × 75 µm ID, BEH 130 material, 1.7 µm particle size) at a flow rate of 350 nl/min. The gradient used for peptide separation was 1–30% B over 13 min, 30–50% B over 5 min, 50–95% B over 5 min, isocratic at 95% B for 4 min, and a return to 1% B over 1 min (phases A and B composed of 0.1% [v/v] FA in water and 0.1% [v/v] FA in 100% acetonitrile, respectively); the analytical column was re-equilibrated for 9 min prior to the next injection. The eluted peptides were transferred via the NanoLockSpray ion source into a Synapt HDMS tandem mass spectrometer (Waters) operated in V-mode with a resolving power of at least 10,000 full width at half height. The source temperature was set to 80°C, cone gas flow 20 L/h, and the nanoelectrospray voltage was 3.2 kV. A 650 fmol/μL human Glu-Fibrinopeptide B in 0.1% aqueous FA/acetonitrile [1:1, v/v] was infused at a flow rate of 0.5 μL min^-1^ through the reference sprayer every 30 seconds to compensate for mass shifts in MS and MS/MS fragmentation mode.

LC-MS data were collected using MassLynx v4.1 software (Waters) under data-independent acquisition that utilizes alternating scanning in low (MS) and elevated energy (MS^E^) mode. In low energy mode, data were collected at constant collision energy of 6 eV. In elevated MS^E^ mode, collision energy was ramped from 15 to 40 eV. MS and MS^E^ data were acquired over 1.5 sec intervals in the mass range of 50-1700 *m*/*z*.

**Data processing**

The acquired data were processed using ProteinLynx Global Server Browser v.2.5.2 software (Waters) using the ion accounting algorithm as [[1](#_ENREF_1)]. For processing of the raw-data following thresholds for low/high energy scan ions and peptide intensity were used: 150, 10, and 750 counts, respectively. The peptide fragment spectra were searched against the *Phaeodactylum tricornutum* database combined with Viridipantae database, both were downloaded on March 3, 2014 from http://www.uniprot.org/. Database searching was restricted to tryptic peptides with a variable carbamidomethyl modification for Cys residues. To investigate possible probe modifications we specified the following variable modifications based on previous findings of Cys Michael adducts with 2,4-decadienal (DD) [[2](#_ENREF_2)], imine formation of Lys with DD [[2](#_ENREF_2),[3](#_ENREF_3)] and DDY [[4](#_ENREF_4)], Lys Michael adduct formation with unsaturated aldehydes [[5](#_ENREF_5),[6](#_ENREF_6)] and imidazole adduct formation between Arg and [4-oxo-2-nonenal](http://www.abcam.com/4-oxo-2-nonenal-4-one-ab120878.html) [[7](#_ENREF_7)]:

- for TAMRA-PUA and arising Michael adducts of Cys or Lys 661.314 Da (named: TAMRA-PUA_Cys_Michaelreaction or TAMRA-PUA_Lys_Michaelreaction),
- for TAMRA-PUA and arising imine formation with Lys or imidazole formation with Arg with loss of H_2_O 643.303 Da (named: TAMRA-PUA_Lys or TAMRA-PUA_Arg),
- for DDY and arising Michael adducts of Cys or Lys 148.089 Da (named: DDY_Cys_Michaelreaction or DDY_Lys_Michaelreaction),
- for DDY and arising imine formation with Lys or imidazole formation with Arg with loss of H_2_O 130.078 Da (named: DDY_Lys or DDY_Arg).

Further, default searching parameter specifying mass measurement accuracy were used, minimum number of product ion matches per peptide (3), minimum number of product ion matches per protein (5), minimum number of peptide matches (1), and maximum number of missed tryptic cleavage sites (2). Maximum false positive rate was set to 4% and all peptides matched under the 4% FDR were considered as correct assignments. For data processing only conclusive proteins were considered.

Proteins of each gel (gel 1, gel 2, gel 3) were classified according to the following results:

- confident target protein: protein found as single hit in an excised gel spot,
- labeled target protein: protein labeled by DDY or TAMRA-PUA in an excised gel spot besides other unlabeled proteins,
- putative target protein: protein found in an excised gel spot with more than one protein hit.

Additional classification into proteins with assigned biological processes or molecular functions or predicted proteins without assignable function was made by using InterPro (http://www.ebi.ac.uk/interpro).

Results of all 3 gels were included in S1 Table and Table 1. Therefore proteins were classified according the following procedure:

- confident target proteins:
- A) target proteins: the protein was found in at least two different gels, at least in one excised gel spot the protein occurred as single hit
- B) target proteins labeled: the protein was found in at least two different gels, at least in one excised gel spot the DDY or TAMRA-PUA labeled protein was identified
- probable target proteins
- C) probable target proteins: the protein was only found in one gel as single hit in an excised gel spot
- D) probable target proteins labeled: the protein was found in only one gel and is labeled by DDY or TAMRA-PUA
- E) probable target proteins: the protein was found in at least two different gels with more than one protein hit per excised gel spot
- putative target proteins
- F) putative target proteins: the protein was found in only one gel with more than one protein hit per excised gel spot.

**References**

1. Li GZ, Vissers JP, Silva JC, Golick D, Gorenstein MV, Geromanos SJ. Database searching and accounting of multiplexed precursor and product ion spectra from the data independent analysis of simple and complex peptide mixtures. Proteomics. 2009 Mar;9(6):1696-719.

2. Zhu X, Tang X, Zhang J, Tochtrop GP, Anderson VE, Sayre LM. Mass spectrometric evidence for the existence of distinct modifications of different proteins by 2(*E*),4(*E*)-decadienal. Chem Res Toxicol. 2010;23(3):467–73.

3. Sigolo CAO, Di Mascio P, Medeiros MHG. Covalent modification of cytochrome *c* exposed to *trans*,*trans*-2,4-decadienal. Chem Res Toxicol. 2007;20(8):1099–110.

4. Wolfram S, Würfel H, Habenicht SH, Lembke C, Richter P, Birckner E, et al. A small azide-modified thiazole-based reporter molecule for fluorescence and mass spectrometric detection. Beilstein J Org Chem. 2014;10:2470–9.

5. Isom AL, Barnes S, Wilson L, Kirk M, Coward L, Darley-Usmar V. Modification of cytochrome *c* by 4-hydroxy- 2-nonenal: evidence for histidine, lysine, and arginine-aldehyde adducts. J Am Soc Mass Spectrom. 2004 Aug;15(8):1136–47.

6. Ichihashi K, Osawa T, Toyokuni S, Uchida K. Endogenous formation of protein adducts with carcinogenic aldehydes: implications for oxidative stress. J Biol Chem. 2001;276(26):23903-13.

7. Oe T, Lee SH, Silva Elipe MV, Arison BH, Blair IA. A novel lipid hydroperoxide-derived modification to arginine. Chem Res Toxicol. 2003 Dec;16(12):1598–605.
